# The lower airways microbiome and antimicrobial peptides in idiopathic pulmonary fibrosis differ from chronic obstructive pulmonary disease

**DOI:** 10.1371/journal.pone.0262082

**Published:** 2022-01-06

**Authors:** Kristel S. Knudsen, Sverre Lehmann, Rune Nielsen, Solveig Tangedal, Ingvild Haaland, Pieter S. Hiemstra, Tomas M. Eagan

**Affiliations:** 1 Department of Clinical Science, University of Bergen, Bergen, Norway; 2 Department of Thoracic Medicine, Haukeland University Hospital, Bergen, Norway; 3 Department of Pulmonology, Leiden University Medical Center, Leiden, Netherlands; University of Maine, UNITED STATES

## Abstract

**Background:**

The lower airways microbiome and host immune response in chronic pulmonary diseases are incompletely understood. We aimed to investigate possible microbiome characteristics and key antimicrobial peptides and proteins in idiopathic pulmonary fibrosis (IPF) and chronic obstructive pulmonary disease (COPD).

**Methods:**

12 IPF patients, 12 COPD patients and 12 healthy controls were sampled with oral wash (OW), protected bronchoalveolar lavage (PBAL) and right lung protected sterile brushings (rPSB). The antimicrobial peptides and proteins (AMPs), secretory leucocyte protease inhibitor (SLPI) and human beta defensins 1 and 2 (hBD-1 & hBD-2), were measured in PBAL by enzyme linked immunosorbent assay (ELISA).

The V3V4 region of the bacterial 16S rDNA gene was sequenced. Bioinformatic analyses were performed with QIIME 2.

**Results:**

hBD-1 levels in PBAL for IPF were lower compared with COPD. The predominant phyla in IPF were *Firmicutes*, *Bacteroides* and *Actinobacteria*; *Proteobacteria* were among top three in COPD. Differential abundance analysis at genus level showed significant differences between study groups for less abundant, mostly oropharyngeal, microbes. Alpha diversity was lower in IPF in PBAL compared to COPD (p = 0.03) and controls (p = 0.01), as well as in rPSB compared to COPD (p = 0.02) and controls (p = 0.04). Phylogenetic beta diversity showed significantly more similarity for IPF compared with COPD and controls. There were no significant correlations between alpha diversity and AMPs.

**Conclusions:**

IPF differed in microbial diversity from COPD and controls, accompanied by differences in antimicrobial peptides. Beta diversity similarity between OW and PBAL in IPF may indicate that microaspiration contributes to changes in its microbiome.

## Introduction

Idiopathic pulmonary fibrosis (IPF) is a progressive and chronic interstitial lung disease; characterized by a rapid decline in lung function, short life expectancy and limited treatment options. The prevalence appears to be increasing and a better understanding of the pathogenesis is needed for the development of novel or improved therapy [1].

Whereas IPF is relatively rare, chronic obstructive pulmonary disease (COPD) is common [2]. Both diseases share smoking as a risk factor, increasing prevalence at higher age and chronic systemic inflammation. Respiratory infections may play a role in the pathogenesis of both diseases, although the extent and nature of their involvement may differ markedly.

The respiratory tract below the vocal cords was considered sterile until recently [3]. High-throughput 16S rDNA sequencing has revealed the presence of a diverse, low-biomass bacterial microbiome in the lungs [4]. A distorted microbiome in the lower airways might be involved in the pathogenesis of IPF and COPD, but the microbiome in both diseases are yet incompletely characterized [5–9].

Antimicrobial peptides and proteins (AMPs) are main effector molecules in innate immunity against respiratory pathogens [10]. There are few studies on AMP levels in respiratory secretions in chronic lung diseases [11–13]. Arguably, one sign of a distorted microbiome would be a corresponding impact on local immunity, which is not fully investigated [7], and no studies exist on AMPs and the lower airways microbiome.

The current gold standard for lower airways sampling of the microbiome is protected sampling with a sterile wax tip catheter inserted during bronchoscopy [14]. Both protected bronchoalveolar lavage (PBAL) and protected sterile brushings (PSB) can be collected this way.

In this study we examined the oral and lower airways microbiome and the secretory leucocyte protease inhibitor (SLPI) and human beta defensins 1 and 2 (hBD-1 & hBD-2) in 12 IPF patients, 12 COPD patients and 12 healthy controls. The aim was to investigate whether the microbiome differed between the study groups and if levels of the immune effector molecules varied, thus indicating a potential impact of the microbiome.

## Methods

### Study design

The study design is an observational case-control study of the stable lower airways microbiome in two disease groups compared with healthy controls. Subjects were enrolled from The Bergen COPD Microbiome study ("MicroCOPD") and The Microbiome in Interstitial Lung Disease study ("MicroILD") at the Department of Thoracic Medicine, Haukeland University Hospital, Norway.

The MicroCOPD Study included 130 COPD patients, 103 healthy controls and 16 asthma patients examined between April 2012 and June 2015. The study design and data collection are published [15]. Patients from the MicroILD study were recruited between December 2014 and December 2016 prior to medical examination including bronchoscopy with PBAL for suspected interstitial lung disease (ILD). Seventy patients were included in the MicroILD study, where the IPF diagnosis was set for 12 patients in multi-disciplinary meetings. From the MicroCOPD study, 12 COPD patients and 12 healthy controls were randomly selected by use of the *runiform ()* function in Stata. One COPD patient used low dose prednisolone upon inclusion, and no participants used antibiotics at least two weeks prior to inclusion.

From all patients we collected blood samples, lung function measurements and performed a standardized interview for medical history, medications and comorbidities.

The MicroCOPD and MicroILD studies were approved by the Norwegian regional ethical committee (REK) with case numbers 2011/1307 & 2014/1393 respectively. All participants were given oral and written information regarding study participation, and all participants provided written informed consent prior to inclusion per the guidelines of the regional ethical committee. The study was partially funded by a grant from the regional health authorities (“Helse-Vest”). The funders had no role in study design, data collection and analysis, decision to publish, or preparation of the manuscript.

### Bronchoscopy

All bronchoscopies were performed in one centre with consistent methodology. The participants were in supine position with oral access and given topical anaesthesia. Light sedation with intravenous alfentanil was offered to all. Suctioning was avoided until passing the carina to minimize contamination. We sampled oral wash (OW), 3 x protected sterile brushes from the right lower lobe (rPSB) and 2 x 50ml PBAL (For IPF 3 x 50ml) through a sterile protective sheath from the right middle lobe. For fluid samples, we used a sterile phosphate-buffered saline (PBS) fluid opened within 24 hours before the procedure. A negative control sample was always collected directly from the PBS fluid bottle without being contaminated by the patient or the bronchoscope.

### BAL measurements

Two cytospin slides were made from BAL fluid from each participant, colored with May-Grunwald/Giemsa staining. 300+ cells were counted from each slide to provide the BAL differential cell counts.

SLPI [16] and hBD-1 (PeproTech, London; UK) & hBD-2 (Antigenix America, Melville, NY, US) were measured in PBAL by enzyme linked immunosorbent assay (ELISA), performed at the Leiden University Medical Center, the Netherlands.

### DNA extraction and 16S sequencing

The detailed protocol for processing is published [17]. Briefly, bacterial DNA was extracted by enzymatic and mechanical lysis methods, using MP Biomedicals’ FastPrep -24 instrument and the FastDNA Spin Kit from MP Biomedicals (LLC, Solon, OH, USA). The V3-V4 region of the 16S rDNA gene was PCR amplified and prepared for paired-end sequencing. After initial PCR, an index PCR was performed, enabling 96 samples in each run. DNA in the samples was quantified and normalized before they were loaded and sequenced according to the protocol for 16S Metagenomic Sequencing Library Preparation for the Illumina MiSeq System (Part # 15044223 Rev. B). All runs included negative control samples of PBS fluid, a generous donor sample similar across all runs, and for a sub-sample a mock-community, thus enabling both assessment of laboratory reagent contamination and calculation of adjusted rates [18].

### Bioinformatics

QIIME 2 [19] was chosen as bioinformatic pipeline. The amplicon sequences were quality filtered using the Divisive Amplicon Denoising Algorithm 2 (DADA2) [20] and PCR-made sequences (chimeras) were removed through DADA2 and VSEARCH [21]. Sequences were clustered into amplicon sequence variants (ASVs) and processed with removal of contaminants with the Decontam package in R, which allows for removing contaminants based on presence in the negative control samples relative to biological samples (*prevalence* method) [22]. A description of contaminants found in the negative controls is provided in the S1 Appendix. Taxonomy was assigned to the ASVs with a trained classifier using the Human Oral Microbiome Database (HOMD) [23] and aligned with mafft [24].

Samples were rarefied to 1000 reads and a phylogenetic tree was constructed using FastTree for diversity analyses. Principle coordinate analyses (PCoA) of weighted UniFrac distances were run in QIIME 2 and exported to R to be plotted using ggpubr.

### Statistical analyses

Stata version 14.2 was used for statistical analyses. Categorical variables were analysed as proportions and continuous variables as means or medians depending on distribution. The differential abundance and its compositionality were tested with Analysis of Composition of Microbes with Bias Correction (ANCOM-BC) [25] in R and ANOVA-like differential expression analysis 2 (ALDEx2) in QIIME 2. Differences in alpha diversity, expressed as Faith’s phylogenetic and Shannon’s non-phylogenetic diversity indexes, were tested with Kruskal-Wallis tests in Stata. Differences in beta diversity, exemplified with phylogenetic weighted and unweighted UniFrac and non-phylogenetic Bray-Curtis, were tested with pairwise PERMANOVA in QIIME 2. Pairwise correlations between the antimicrobial peptides and 1) the relative abundance of the two most common phyla and three most common genera, 2) alpha diversity, and 3) lung function were tested with Spearman’s p. For all analyses, differences or correlations with a p-value < 0.05 were considered significant.

## Results

The demographics of the participants are outlined in Table 1. Lung function varied between the study groups as expected, and the IPF patients were predominantly male with higher age compared to COPD patients and controls.

**Table 1:** Characteristics of the study population.

	IPF	COPD	Controls	IPF vs COPD	IPF vs Controls	COPD vs Controls
	n = 12	n = 12	n = 12	p*	p*	p*
***Sex*, *%***				0.04	0.41	0.21
Women	33	75	50			
Men	67	25	50			
***Age*, *mean years (SD)***	73.2 (11.1)	65.7 (7.6)	66.4 (7.7)	0.07	0.10	0.82
***Smoking*, *%***				0.02	0.33	0.01
Never	33	0	17			
Ex	58	50	83			
Current	9	50	0			
***Pulmonary function*, *mean % of predicted (SD)***						
FVC	74 (15.6)	96 (21.8)	109 (14.0)	0.01	<0.01	0.09
FEV_1_	77 (11.7)	57 (16.1)	100 (10.8)	<0.01	<0.01	<0.01
DLCO	55 (14.9)	63 (25.5)	103 (12.3)	0.45	<0.01	<0.01

* p for sex and smoking tested by Pearson chi square test, and age and lung function tested by ANOVA.

PBAL measurements are shown for each study group in Table 2. IPF patients had significantly higher percentage of neutrophils and lower percentage of eosinophils in PBAL than COPD patients and controls, as expected. In IPF patients, hBD-1 was significantly lower in PBAL compared with COPD patients (p<0.01) and trending lower than for controls. Human beta defensin-2 was higher in IPF patients than in controls (p = 0.01) and trending lower than for COPD patients (p = 0.06). SLPI in PBAL did not differ significantly, but there was a trend towards lower levels in IPF patients compared to COPD patients and controls.

**Table 2:** BAL cell counts and antimicrobial peptides in BAL in the three study groups.

	IPF	COPD	Controls	IPF vs COPD	IPF vs Controls	COPD vs Controls
	n = 12	n = 12	n = 12	p*	p*	p*
***BAL cell content %*, *mean (SD*** ¶ ***)***						
Macrophages	72.4 (25.0)	86.2 (5.8)	78.9 (12.1)	0.08	0.44	0.07
Neutrophils	11.8 (10.2)	4.4 (2.7)	5.1 (3.7)	0.02	0.047	0.57
Lymphocytes	15.6 (24.8)	8.7 (4.6)	15.3 (11.1)	0.35	0.97	0.07
Eosinophils	0.1 (0)	0.8 (1.1)	0.7 (0.7)	<0.01	<0.01	0.93
***Antimicrobial peptides in BAL*, *median (IQR*** ¶ ***)***						
SLPI** ng/ml	136 (106–235)	196 (148–299)	184 (153–274)	0.20	0.10	0.89
hBD-1*** pg/ml	205 (136–254)	606 (371–692)	403 (170–503)	<0.01	0.08	0.15
hBD-2*** pg/ml	116 (10–171)	10 (10–58)	10 (10–10)	0.06	0.01	0.47

* p for BAL cell counts tested by ANOVA and for antimicrobial peptides tested by Kruskal-Wallis test.

** Secretory leucocyte protease inhibitor (SLPI).

***human beta defensins 1 and 2 (hBD-1 & hBD-2). Lower limit of detection for hBD-2 was 10 pg/ml.

¶ Interquartile range (IQR), Standard deviation (SD).

The correlations between levels of AMPs in BAL and lung function, smoking, and use of inhaled steroids (COPD only), is presented in the S1 Table. Among 48 associations tested, only 3 was found statistically significant.

### Taxonomy

**Fig 1:**
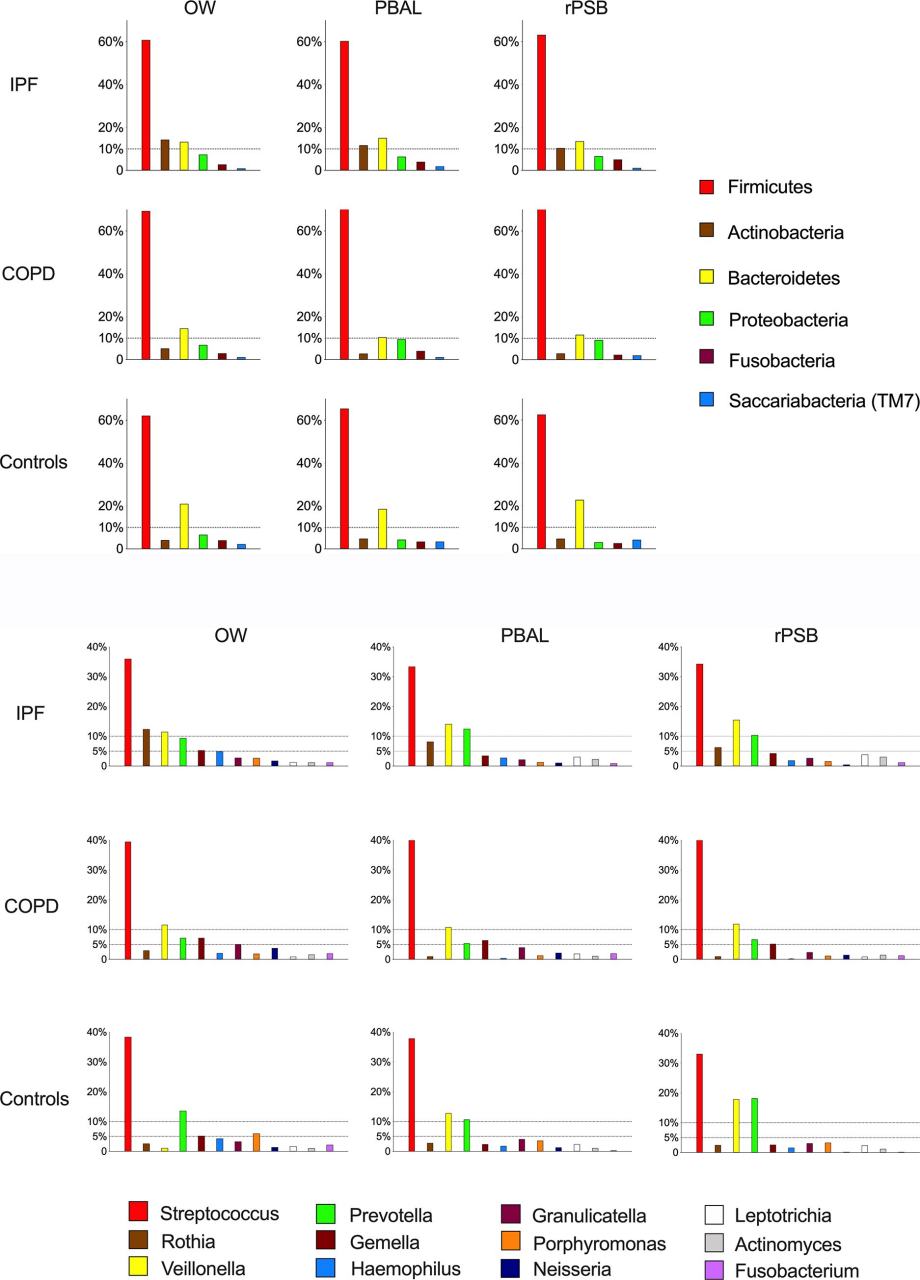
**a**. Bacterial taxonomy at the phylum level by study groups (IPF: Idiopathic pulmonary fibrosis, COPD: Chronic obstructive pulmonary disease, controls) and sample types (OW: Oral wash, PBAL: Protected bronchoalveolar lavage, rPSB: Right protected sterile brushes). Each bar represents one taxon and is visualised in the order of decreasing relative abundance in OW for IPF patients. **b**. Bacterial taxonomy at the genus level by study groups (IPF: Idiopathic pulmonary fibrosis, COPD: Chronic obstructive pulmonary disease, controls) and sample types (OW: Oral wash, PBAL: Protected bronchoalveolar lavage, rPSB: Right protected sterile brushes). Each bar represents one taxon and is visualised in the order of decreasing relative abundance in OW for IPF patients.

The taxonomic distribution by study groups for the different sample types is illustrated with rank abundance plots at phylum and genus levels in Fig 1A and 1B respectively. Each bar represents one taxon and is visualised in the order of decreasing relative abundance in OW for IPF patients. The predominant phyla found in IPF patients were *Firmicutes*, *Actinobacteria and Bacteroidetes*; whereas *Proteobacteria* was the third most predominant in COPD patients (Fig 1A). The relative abundance of the 12 most common genera displays the dominance of *Streptococci* for all study groups, followed by *Rothia*, *Veillonella* and *Prevotella* for IPF patients (Fig 1B).

Differential abundance analysis with ANCOM-BC at phylum level showed no statistically significant differences between the study groups, but there were several significant results at genus level, with infrequent microbes (Table 3).

**Table 3:** Differentially abundant phyla or genera found by differential abundance testing.

Test	Sample Type	Level	Taxonomic results
ANCOM-BC	OW	Phyla	No differential abundant phyla
		Genera	**IPF vs COPD**: Bacteroidetes[G-5], Peptostreptococcaceae[XI][G-1], Kingella
			**IPF vs Control**: Mogibacterium, Mycoplasma
			**COPD vs Control**: Peptostreptococcaceae[XI][G-1], Mycoplasma, Kingella
	PBAL	Phyla	No differential abundant phyla
		Genera	**IPF vs COPD:** Alloscardovia, Bacteriodales [g-2], Peptoniphilus, Mogibacterium, Moraxella
			**IPF vs Control**: Lactobacillus, Mogibacterium
			**COPD vs Control**: Filifactor, Moraxella
	rPSB	Phyla	No differential abundant phyla
		Genera	**IPF vs COPD**: Corynebacterium, Segetibacter, Lactobacillus, Lautropia
			**IPF vs Control**: Corynebacterium, Segetibacter, Lactobacillus, Lautropia
			**COPD vs Control**: *none*
ALDEx2	OW	Phyla	No differential abundant phyla
		Genera	No differential abundant genera
	PBAL	Phyla	No differential abundant phyla
		Genera	No differential abundant genera
	rPSB	Phyla	No differential abundant phyla
		Genera	No differential abundant genera

No significant differences were found in the differential abundance of genera or phyla between the study groups for any sample types when performing ALDEx2 analysis (Table 3).

**Fig 2:**
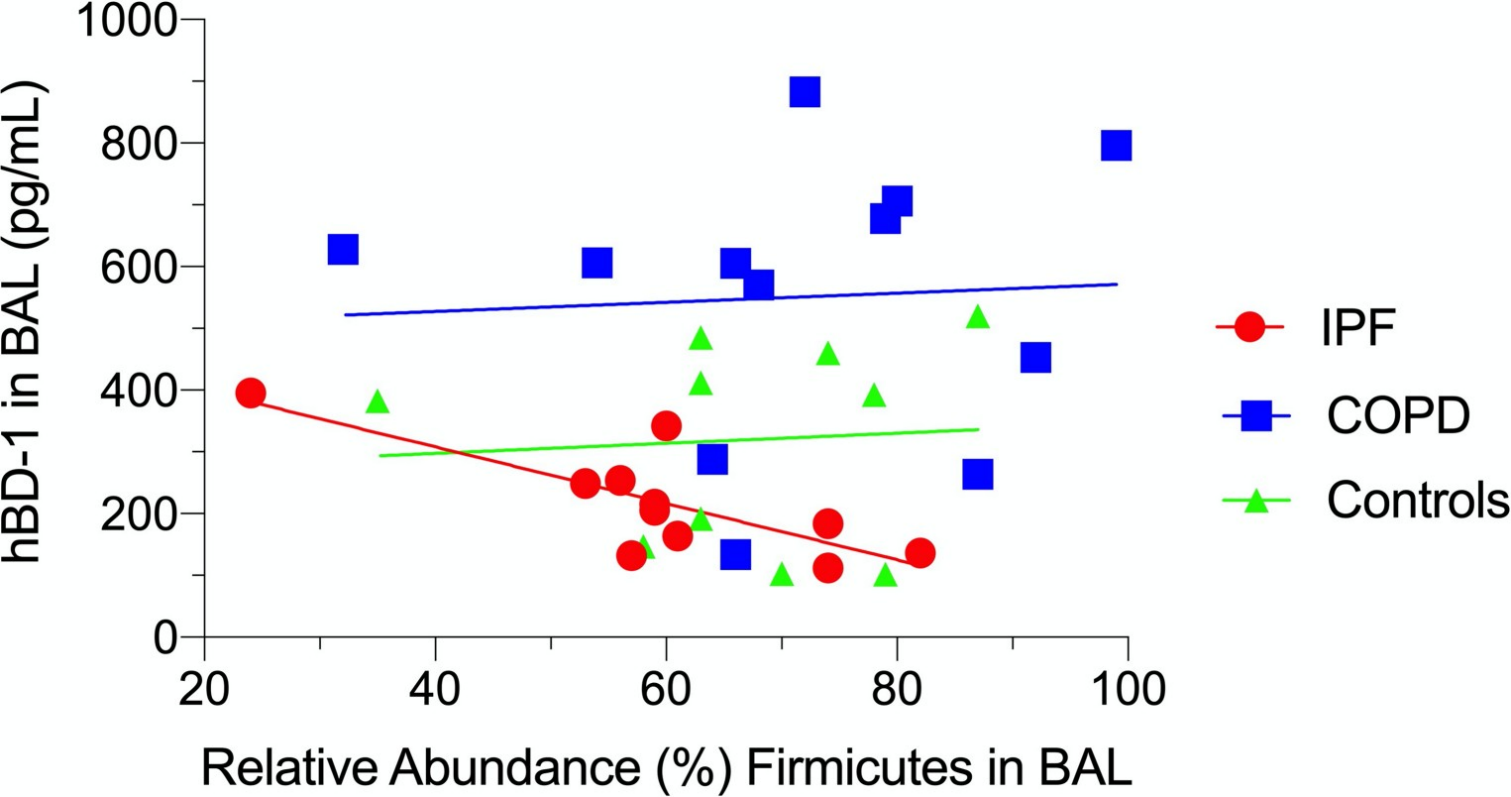
The relationship between levels of human beta defensin 1 (hBD-1) and the relative abundance of *Firmicutes* in BAL fluid in IPF patients, COPD patients and controls.

Testing the 90 pairwise correlations between lung function, the measured levels of SLPI, hBD-1 & hBD-2, and the relative abundances of the most common phyla and genera, stratified by study group, 5 were statistically significant for lung function, and 4 were statistically significant for the AMPs (S2 Table). Three of each of the significant correlations were in the IPF group, where notably hBD-1 was negatively correlated with the relative abundance of *Firmicutes* (p = 0.03, correlation coefficient -0.64), an association driven by the abundance of *Streptococci* (p = 0.007, correlation coefficient -0.80). This association was not seen for COPD patients and controls (Fig 2).

### Diversity

**Fig 3:**
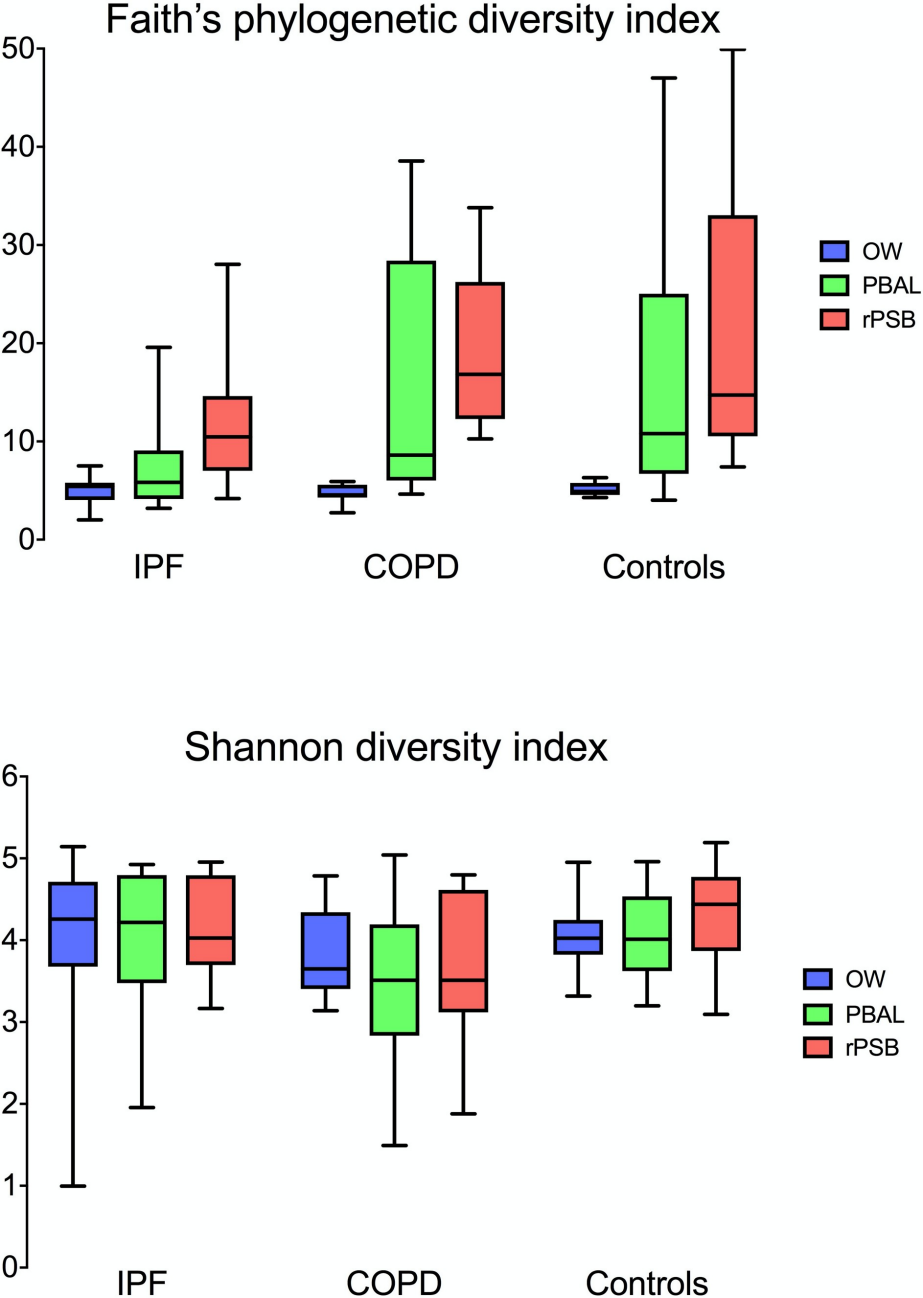
Faith’s phylogenetic diversity and Shannon’s non-phylogenetic diversity by study groups (IPF: Idiopathic pulmonary fibrosis, COPD: Chronic obstructive pulmonary disease, controls) and sample types (OW: Oral wash, PBAL: Protected bronchoalveolar lavage, rPSB: Right protected sterile brushes) illustrated with boxplots. Faith’s phylogenetic diversity was significantly lower in IPF patients for PBAL compared to COPD patients (p = 0.03) and controls (p = 0.01), and in rPSB compared to COPD patients (p = 0.02) and controls (p = 0.04). No significant results for Shannon’s non-phylogenetic diversity.

The distribution of alpha diversity is visualized in Fig 3 by study groups and sample types. Faith’s phylogenetic diversity was significantly lower in IPF patients in both PBAL compared to COPD patients (p = 0.03) and controls (p = 0.01), and in rPSB compared to COPD patients (p = 0.02) and controls (p = 0.04). No differences between study groups were found for OW. No differences were found with Shannon’s non-phylogenetic diversity. There were no significant correlations between either metric of alpha diversity and SLPI, hBD-1, or hBD-2 using Spearman’s *p* (S3 Table).

**Fig 4:**
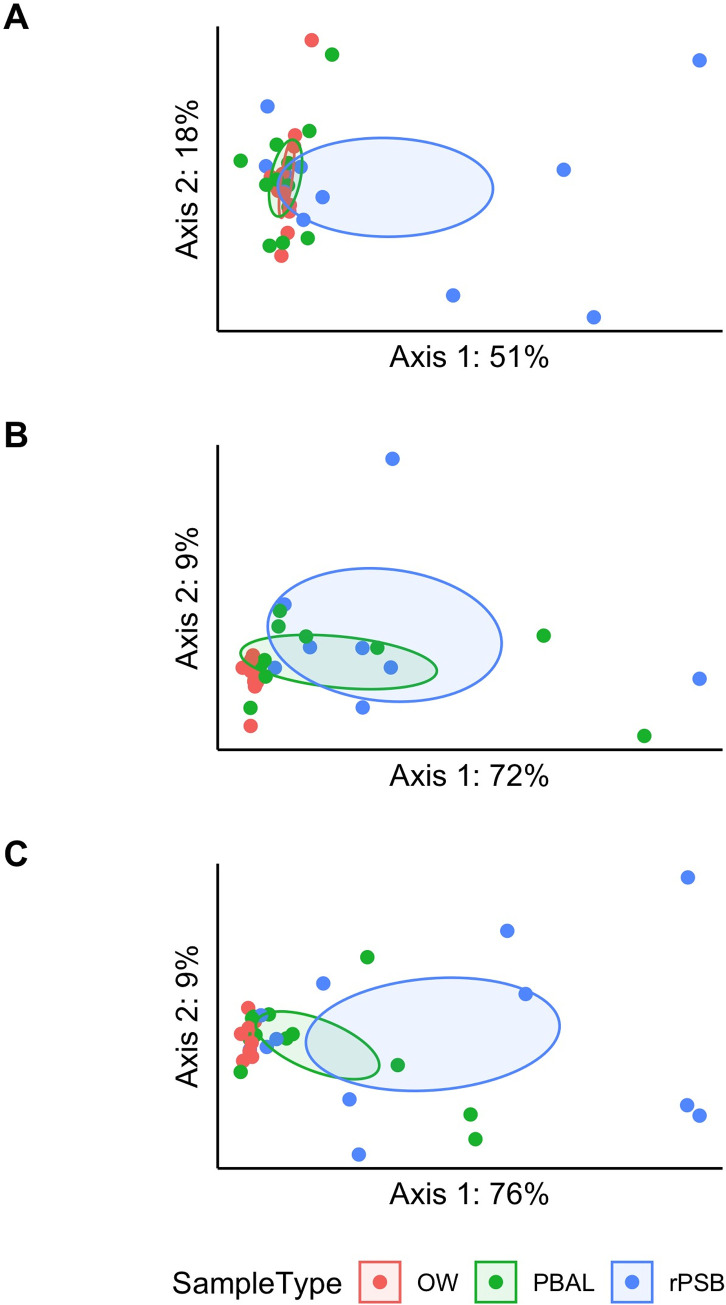
Principal coordinates analysis (PCoA) of weighted UniFrac (beta diversity). PERMANOVA (999 permutations) for the underlying distance matrices showed significant more similar beta diversity among the sample types for IPF (Idiopathic pulmonary fibrosis) patients illustrated by the blue dot OW (oral wash), green dot PBAL (protected bronchoalveolar lavage) and red dot rPSB (right protected sterile brushes) compared to COPD (Chronic obstructive pulmonary disease) and controls.

The principal coordinates analysis (PCoA) plots for weighted UniFrac (Fig 4) show oral samples separate from bronchial samples to a lesser degree in IPF patients compared to COPD and controls. This was confirmed statistically by PERMANOVA tests (S4 Table).

Further exploration of the PCoA analyses with loading plots are presented in the S1 Appendix.

## Discussion

This study has demonstrated that the lower airways microbiome and levels of lower airways antimicrobial peptides in IPF patients differed from those in COPD patients and controls. Compared to COPD patients and controls, IPF patients exhibited less phylogenetic alpha diversity in their microbiome of the lower airways and a more similar phylogenetic beta diversity between oral and lower airways. The differences in the lower airways microbiome between IPF patients and the other study groups were mirrored by changes in levels of the antimicrobial peptides. In IPF patients, hBD-1 was lower compared to COPD patients and hBD-2 was higher compared to controls. Although there was no statistically significant association between alpha diversity and the AMPs, hBD-1 was negatively correlated with the relative abundance of *Firmicutes*, in contrast with COPD patients and controls.

Only a few studies on chronic pulmonary diseases have performed analysis of AMPs in BAL. These peptides are produced in the lung and other mucosal tissues, as well as by immune and inflammatory cells, and play an important role in antimicrobial activity, immune modulation and wound repair [10]. Inflammatory markers might have a stronger signal in BAL fluid compared to blood. Hollander et al [13] found significantly higher values for SLPI and IL-8 in BAL concentrations compared to plasma, suggesting predominant production in lung tissue. In our study, IPF patients had lower levels of SLPI and hBD-1 in PBAL compared to COPD patients and controls. We have previously shown that SLPI was lower in sputum during COPD exacerbations compared to in stable COPD [11] and neutrophilic inflammation has been associated with SLPI deficiency or inactivation [12]. Thus, lower levels of SLPI and hBD-1 could be an effect of the increased neutrophilic inflammation seen in the IPF group, or merely impaired wound repair, altered microbiome and higher disease burden. Of note, neutrophil counts in PBAL are inversely correlated with FVC and thus a potential marker of disease activity [26]. Also TGF-β activity in the lungs of IPF patients could have contributed to these lower levels [27].

The airway mucosal surfaces contain a diversity of microbes whose composition differs between patients with pulmonary diseases and healthy controls. A double-blind multicentre study with 181 patients randomised to receive co-trimoxazole or placebo had no effect on lung function, but resulted in improved quality of life and mortality reduction, possibly due to the antimicrobial and immunomodulatory effects of antibiotics [28]. Han et al [29] evaluated the lung microbiome in stable IPF and linked microbial composition and bacterial burden with disease outcome. They found operational taxonomic units (OTUs) belonging to either the *Streptococcus* or *Staphylococcus* genus to be associated with increased risk of disease progression. Although we do not know whether a distorted microbiome in IPF patients is a cause or a consequence of IPF, it cannot be ruled out as a predictor for disease progression.

In the healthy lung, the most common bacteria described in the lower airways thus far are similar to those in the upper airways, and are dominated by the phyla *Firmicutes*, *Bacteroidetes* and *Proteobacteria*, and the genera *Prevotella*, *Veillonella* and *Streptococcus* [14,30].

In our study, the most abundant phyla (*Firmicutes* and *Bacteroidetes*) were shared by IPF and COPD patients, while *Actinobacteria* and *Proteobacteria* were the third most abundant phyla in the respective study groups. This is coherent with other studies [6,31–34]. The most abundant genera in our study were *Streptococcus*, *Rothia*, *Veillonella* and *Prevotella*, which are described earlier for IPF patients, but also in other chronic pulmonary diseases [29,35]. The distribution of the most abundant genera appeared somewhat dissimilar in the rank abundance plots, but were not found to be significantly different in the differential abundance analyses.

We used various statistical tests to explore the compositionality of the data. ALDEx2 found no phyla or genus with statistically significant abundance between study groups, but the newer and perhaps more robust algorithm, ANCOM-BC, found several genera, which differed significantly between IPF patients and the other study groups. Neither of these were among the 20 most abundant genera overall, thus raising the question as to which degree this is clinically relevant. Interestingly though, several of these genera likely originate from the oropharynx, where some are linked to periodontal diseases (*Mogibacterium*, *Filifactor*, *Lactobacillus*) [36]. One possible interpretation is that IPF patients are more vulnerable to changes in airway microbiota as a result of microaspiration of pathogenic oral microbes than healthy controls and stable COPD patients. The finding of more similarity between oral and bronchial samples for IPF patients than for COPD patients and controls is in line with this hypothesis.

Microbial diversity is another important factor to examine when assessing the microbiome. In our study there were several statistically significant differences in diversity. Lower phylogenetic alpha diversity in IPF patients is in line with some previous studies. In a study of 65 IPF patients and 44 control subjects, Molyneaux et al [35] demonstrated that the microbial communities of subjects with IPF were less diverse compared to healthy controls. Takahashi et al [34] found that alpha diversity was smaller in a IPF progression group than in the non-progression group, suggesting the diversity of the pulmonary microbiome could be used as a prognostic indicator. O’Dwyer et al [37] showed that decreased alpha diversity of lung microbiota in IPF patients was significantly associated with increased alveolar concentrations of cytokines, indicating a possible immune-microbiome interaction. However much remains to be discovered regarding the microbiome on alveolar surfaces.

The similar beta diversity in the oral and lower airways in IPF patients in our study could indicate that microaspiration is a pathogenic factor. Several studies indicate that the lung microbiome is influenced by silent microaspiration from the oropharynx [30,31,38]. Since gastroesophageal reflux disease (GERD) has an estimated prevalence of up to 90% in IPF patients [39], this is highly relevant.

Some studies have described that increased lung bacterial burden in IPF patients predict subsequent disease progression [35,37,40]. GERD and microaspiration are possible sources of an increased bacterial burden. The finding of lower alpha diversity, differences in taxa composition linked to oral taxa, and a different local immune signature in IPF patients could perhaps all be consequences of these mechanisms. Due to the resembling upper and lower airways microbiota, microbial samples of the oral flora in IPF patients could possibly be important predictors of disease progression in itself, with the advantage of being vastly simpler to collect and measure.

Sampling low biomasses is challenging. The density of the bacterial DNA in the upper airways is at least 100-fold higher than in the lower airways [38]. In order to minimize contamination from the upper airways, we standardized protected sampling. However, there are some methodological limitations to address. First, there was a slight difference in PBAL volume collected between the study groups as described in the methodology chapter. Whether this affects the dilution of the measured markers is possible, however BAL yield differs between subjects invariably and we measured concentrations and relative abundances. Thus, this should not affect the interpretation of lung microbiota data. Relative abundance data are compositional and bacterial DNA burden varies more between specimens than dilution can affect [41]. An increased fraction of neutrophils in PBAL in IPF patients was in line with previous studies [42], indicating representative BAL cell compositionality. Second, collecting PBAL from one lobe might not be fully representative for the disease examined, but intra-subject variations are less than inter-subject variation when studying the bacterial communities [43]. Third, low biomass samples are vulnerable to contamination from multiple sources during sampling and laboratory processing [44]. A newly published paper describing the use and implications with the validated Decontam method in the MicroCOPD study helped us identify contaminants [45]. Fourth, the sample size was limited and therefore we could not adjust for all variables within a category. Several confounders could have influenced our results, including smoking, lung function and medication use. Fifth, the exploratory nature of the study, and lack of standardized effect size measures for differences in taxonomy, prevented us from performing a priori sample size calculations. Finally, this is a cross-sectional study and consequently there is need for longitudinal studies to follow microbiota alterations over time and conclude on its consequences for disease development.

The role of the microbiome in pulmonary chronic diseases is incompletely understood. This study has demonstrated that the lower airways microbiome differed between IPF patients, COPD patients and healthy controls. IPF patients demonstrated lower diversity in the lower airways, associated with differences in levels of the AMPs, indicating that the microbiome is either impacting the immune system or vice versa. There are limited treatment options and the impact of novel classes of antifibrotic drugs on the microbiome in IPF is unknown. Future research on modifying the microbiome in IPF patients may be a worthwhile effort in searching for treatments preventing IPF progression.

## Supporting information

S1 Table Spearman’s correlation coefficients for the association between the levels of antimicrobial peptides in BAL and lung function. The association with smoking and inhaled steroid use (COPD only) was explored by Kruskal-Wallis tests.
(DOCX)

S2 Table Spearman’s correlation coefficients and p-values between the lung function and the three AMPs stratified by study group, and the relative abundance (%) of the two most common phyla and three most common genera. For the categorical variables smoking (never, ex, current), and use of inhaled steroids (in COPD only), the correlation was assessed by Kruskal-Wallis tests, and p-values provided.
(DOCX)

S3 Table Spearman’s correlation coefficients for the association between alpha diversity assessed by Faith’s phylogenetic and Shannon’s non-phylogenetic to the antimicrobial peptides for the different study groups and all subjects. (DOCX)

S4 Table Beta diversity p-values between study groups for different sampling methods (4a), and between different sampling methods between study groups (4b). (DOCX)

S1 Appendix Supporting information for the article: "The lower airways microbiome and antimicrobial peptides in idiopathic pulmonary fibrosis differ from chronic obstructive pulmonary diseases. (PDF)

## References

[1] Lederer DJ, Martinez FJ (2018) Idiopathic Pulmonary Fibrosis. N Engl J Med 378: 1811–1823. doi: 10.1056/NEJMra1705751 29742380

[2] Global Initiative for Chronic Obstructive Lung Disease. Global strategy for the diagnosis, management, and prevention of chronic obstructive pulmonary disease. www.goldcopdorg/2021-gold-reports/ 2021 Report: 1–164.

[3] Dickson RP, Erb-Downward JR, Martinez FJ, Huffnagle GB (2016) The Microbiome and the Respiratory Tract. Annu Rev Physiol 78: 481–504. doi: 10.1146/annurev-physiol-021115-105238 26527186 PMC4751994

[4] Dickson RP, Erb-Downward JR, Huffnagle GB (2013) The role of the bacterial microbiome in lung disease. Expert Rev Respir Med 7: 245–257. doi: 10.1586/ers.13.24 23734647 PMC4007100

[5] Erb-Downward JR, Thompson DL, Han MK, Freeman CM, McCloskey L, Schmidt LA et al. (2011) Analysis of the lung microbiome in the “healthy” smoker and in COPD. PLoS One 6: e16384. doi: 10.1371/journal.pone.0016384 21364979 PMC3043049

[6] Leiten EO, Nielsen R, Wiker HG, Bakke PS, Martinsen EMH, Drengenes C, et al. (2020) The airway microbiota and exacerbations of COPD. ERJ Open Res 6. doi: 10.1183/23120541.00168-2020 32904583 PMC7456643

[7] O’Dwyer DN, Dickson RP, Moore BB (2016) The Lung Microbiome, Immunity, and the Pathogenesis of Chronic Lung Disease. J Immunol 196: 4839–4847. doi: 10.4049/jimmunol.1600279 27260767 PMC4894335

[8] Spagnolo P, Molyneaux PL, Bernardinello N, Cocconcelli E, Biondini D, Fracasso F, et al. (2019) The Role of the Lung’s Microbiome in the Pathogenesis and Progression of Idiopathic Pulmonary Fibrosis. Int J Mol Sci 20. doi: 10.3390/ijms20225618 31717661 PMC6888416

[9] Sze MA, Dimitriu PA, Suzuki M, McDonough JE, Campbell JD, Brothers JF, et al. (2015) Host Response to the Lung Microbiome in Chronic Obstructive Pulmonary Disease. Am J Respir Crit Care Med 192: 438–445. doi: 10.1164/rccm.201502-0223OC 25945594 PMC4595667

[10] Hiemstra PS, Amatngalim GD, van der Does AM, Taube C (2016) Antimicrobial Peptides and Innate Lung Defenses: Role in Infectious and Noninfectious Lung Diseases and Therapeutic Applications. Chest 149: 545–551. doi: 10.1378/chest.15-1353 26502035

[11] Persson LJ, Aanerud M, Hardie JA, Miodini Nilsen R, Bakke PS, Eagan TM, et al. (2017) Antimicrobial peptide levels are linked to airway inflammation, bacterial colonisation and exacerbations in chronic obstructive pulmonary disease. Eur Respir J 49. 10.1183/13993003.01328-2016 28298400

[12] Parameswaran GI, Sethi S, Murphy TF (2011) Effects of bacterial infection on airway antimicrobial peptides and proteins in COPD. Chest 140: 611–617. doi: 10.1378/chest.10-2760 21349930 PMC3204796

[13] Hollander C, Sitkauskiene B, Sakalauskas R, Westin U, Janciauskiene SM (2007) Serum and bronchial lavage fluid concentrations of IL-8, SLPI, sCD14 and sICAM-1 in patients with COPD and asthma. Respir Med 101: 1947–1953. doi: 10.1016/j.rmed.2007.04.010 17574828

[14] Grønseth R, Drengenes C, Wiker HG, Tangedal S, Xue Y, Husebø GR, et al. (2017) Protected sampling is preferable in bronchoscopic studies of the airway microbiome. ERJ Open Res 3. doi: 10.1183/23120541.00019-2017 28875147 PMC5576223

[15] Grønseth R, Haaland I, Wiker HG, Martinsen EM, Leiten EO, Husebø G, et al. (2014) The Bergen COPD microbiome study (MicroCOPD): rationale, design, and initial experiences. Eur Clin Respir J 1. 10.3402/ecrj.v1.26196 PMC4629717 26557236

[16] van Wetering S, van der Linden AC, van Sterkenburg MA, Rabe KF, Schalkwijk J, Hiemstra PS (2000) Regulation of secretory leukocyte proteinase inhibitor (SLPI) production by human bronchial epithelial cells: increase of cell-associated SLPI by neutrophil elastase. J Investig Med 48: 359–366. 10979241

[17] Hoang T, Wiker H., Eagan T. M. & Drengenes C. 16S Amplicon PCR for the V3-V4 region for the MicroCOPD samples. Published protocol version 1, 1–16 (2019) doi: 10.17504/protocols.io.2sygefw

[18] Drengenes C, Eagan TML, Haaland I, Wiker HG, Nielsen R (2021) Exploring protocol bias in airway microbiome studies: one versus two PCR steps and 16S rRNA gene region V3 V4 versus V4. BMC Genomics 22: 3. doi: 10.1186/s12864-020-07252-z 33397283 PMC7784388

[19] Bolyen E, Rideout JR, Dillon MR, Bokulich NA, Abnet CC, Al-Ghalith GA, et al. (2019) Reproducible, interactive, scalable and extensible microbiome data science using QIIME 2. Nat Biotechnol 37: 852–857. doi: 10.1038/s41587-019-0209-9 31341288 PMC7015180

[20] Callahan BJ, McMurdie PJ, Rosen MJ, Han AW, Johnson AJ, Holmes SP (2016) DADA2: High-resolution sample inference from Illumina amplicon data. Nat Methods 13: 581–583. doi: 10.1038/nmeth.3869 27214047 PMC4927377

[21] Rognes T, Flouri T, Nichols B, Quince C, Mahé F (2016) VSEARCH: a versatile open source tool for metagenomics. PeerJ 4: e2584. doi: 10.7717/peerj.2584 27781170 PMC5075697

[22] Davis NM, Proctor DM, Holmes SP, Relman DA, Callahan BJ (2018) Simple statistical identification and removal of contaminant sequences in marker-gene and metagenomics data. Microbiome 6: 226. doi: 10.1186/s40168-018-0605-2 30558668 PMC6298009

[23] Chen T, Yu WH, Izard J, Baranova OV, Lakshmanan A, Dewhirst FE (2010) The Human Oral Microbiome Database: a web accessible resource for investigating oral microbe taxonomic and genomic information. Database (Oxford) 2010: baq013. doi: 10.1093/database/baq013 20624719 PMC2911848

[24] Katoh K, Misawa K, Kuma K, Miyata T (2002) MAFFT: a novel method for rapid multiple sequence alignment based on fast Fourier transform. Nucleic Acids Res 30: 3059–3066. doi: 10.1093/nar/gkf436 12136088 PMC135756

[25] Lin H, Peddada SD (2020) Analysis of compositions of microbiomes with bias correction. Nat Commun 11: 3514. doi: 10.1038/s41467-020-17041-7 32665548 PMC7360769

[26] Ashitani J, Mukae H, Taniguchi H, Ihi T, Kadota J, Kohno S, et al. (1999) Granulocyte-colony stimulating factor levels in bronchoalveolar lavage fluid from patients with idiopathic pulmonary fibrosis. Thorax 54: 1015–1020. doi: 10.1136/thx.54.11.1015 10525561 PMC1745399

[27] Schrumpf JA, Ninaber DK, van der Does AM, Hiemstra PS (2020) TGF-β1 Impairs Vitamin D-Induced and Constitutive Airway Epithelial Host Defense Mechanisms. J Innate Immun 12: 74–89. doi: 10.1159/000497415 30970352 PMC6959102

[28] Shulgina L, Cahn AP, Chilvers ER, Parfrey H, Clark AB, Wilson EC, et al. (2013) Treating idiopathic pulmonary fibrosis with the addition of co-trimoxazole: a randomised controlled trial. Thorax 68: 155–162. doi: 10.1136/thoraxjnl-2012-202403 23143842

[29] Han MK, Zhou Y, Murray S, Tayob N, Noth I, Lama VN, et al. (2014) Lung microbiome and disease progression in idiopathic pulmonary fibrosis: an analysis of the COMET study. Lancet Respir Med 2: 548–556. doi: 10.1016/S2213-2600(14)70069-4 24767767 PMC4142525

[30] Invernizzi R, Lloyd CM, Molyneaux PL (2020) Respiratory microbiome and epithelial interactions shape immunity in the lungs. Immunology 160: 171–182. doi: 10.1111/imm.13195 32196653 PMC7218407

[31] Dickson RP, Erb-Downward JR, Freeman CM, McCloskey L, Falkowski NR, Huffnagle GB, et al. (2017) Bacterial Topography of the Healthy Human Lower Respiratory Tract. mBio 8. doi: 10.1128/mBio.02287-16 28196961 PMC5312084

[32] Invernizzi R, Wu BG, Barnett J, Ghai P, Kingston S, Hewitt RJ, et al. (2020) The Respiratory Microbiome in Chronic Hypersensitivity Pneumonitis is Distinct from that of Idiopathic Pulmonary Fibrosis. Am J Respir Crit Care Med. 10.1164/rccm.202002-0460OC PMC7874329 32692582

[33] Pragman AA, Kim HB, Reilly CS, Wendt C, Isaacson RE (2012) The lung microbiome in moderate and severe chronic obstructive pulmonary disease. PLoS One 7: e47305. doi: 10.1371/journal.pone.0047305 23071781 PMC3469539

[34] Takahashi Y, Saito A, Chiba H, Kuronuma K, Ikeda K, Kobayashi T, et al. (2018) Impaired diversity of the lung microbiome predicts progression of idiopathic pulmonary fibrosis. Respir Res 19: 34. doi: 10.1186/s12931-018-0736-9 29486761 PMC6389110

[35] Molyneaux PL, Cox MJ, Willis-Owen SA, Mallia P, Russell KE, Russell AM, et al. (2014) The role of bacteria in the pathogenesis and progression of idiopathic pulmonary fibrosis. Am J Respir Crit Care Med 190: 906–913. doi: 10.1164/rccm.201403-0541OC 25184687 PMC4299577

[36] Patini R, Staderini E, Lajolo C, Lopetuso L, Mohammed H, Rimondini L, et al. (2018) Relationship between oral microbiota and periodontal disease: a systematic review. Eur Rev Med Pharmacol Sci 22: 5775–5788. doi: 10.26355/eurrev_201809_15903 30280756

[37] O’Dwyer DN, Ashley SL, Gurczynski SJ, Xia M, Wilke C, Falkowski NR, et al. (2019) Lung Microbiota Contribute to Pulmonary Inflammation and Disease Progression in Pulmonary Fibrosis. Am J Respir Crit Care Med 199: 1127–1138. doi: 10.1164/rccm.201809-1650OC 30789747 PMC6515865

[38] Bassis CM, Erb-Downward JR, Dickson RP, Freeman CM, Schmidt TM, Young VB, et al. (2015) Analysis of the upper respiratory tract microbiotas as the source of the lung and gastric microbiotas in healthy individuals. mBio 6: e00037. doi: 10.1128/mBio.00037-15 25736890 PMC4358017

[39] Lee JS, Collard HR, Raghu G, Sweet MP, Hays SR, Campos GM, et al. (2010) Does chronic microaspiration cause idiopathic pulmonary fibrosis. Am J Med 123: 304–311. doi: 10.1016/j.amjmed.2009.07.033 20362747 PMC2851633

[40] Invernizzi R, Barnett J, Rawal B, Nair A, Ghai P, Kingston S, et al. (2020) Bacterial burden in the lower airways predicts disease progression in idiopathic pulmonary fibrosis and is independent of radiological disease extent. Eur Respir J 55: doi: 10.1183/13993003.01519-2019 31980496 PMC7136009

[41] Dickson RP, Erb-Downward JR, Prescott HC, Martinez FJ, Curtis JL, Lama VN, et al. (2014) Analysis of culture-dependent versus culture-independent techniques for identification of bacteria in clinically obtained bronchoalveolar lavage fluid. J Clin Microbiol 52: 3605–3613. doi: 10.1128/JCM.01028-14 25078910 PMC4187760

[42] Pesci A, Ricchiuti E, Ruggiero R, De Micheli A (2010) Bronchoalveolar lavage in idiopathic pulmonary fibrosis: what does it tell us. Respir Med 104 Suppl 1: S70–3. doi: 10.1016/j.rmed.2010.03.019 20471812

[43] Dickson RP, Erb-Downward JR, Freeman CM, McCloskey L, Beck JM, Huffnagle GB, et al. (2015) Spatial Variation in the Healthy Human Lung Microbiome and the Adapted Island Model of Lung Biogeography. Ann Am Thorac Soc 12: 821–830. doi: 10.1513/AnnalsATS.201501-029OC 25803243 PMC4590020

[44] Eisenhofer R, Minich JJ, Marotz C, Cooper A, Knight R, Weyrich LS (2019) Contamination in Low Microbial Biomass Microbiome Studies: Issues and Recommendations. Trends Microbiol 27: 105–117. doi: 10.1016/j.tim.2018.11.003 30497919

[45] Drengenes C, Wiker HG, Kalananthan T, Nordeide E, Eagan TML, Nielsen R (2019) Laboratory contamination in airway microbiome studies. BMC Microbiol 19: 187. doi: 10.1186/s12866-019-1560-1 31412780 PMC6694601

