# Supplementary material for: The lower airways microbiome and antimicrobial peptides in idiopathic pulmonary fibrosis differ from chronic obstructive pulmonary disease
Source: PLoS One. 2022 Jan 6;17(1):e0262082. doi: 10.1371/journal.pone.0262082 (PMC8735599; doi:10.1371/journal.pone.0262082)
Supplement: S1 Table — The association with smoking and inhaled steroid use (COPD only) was explored by Kruskal-Wallis tests. (DOCX) [file pone.0262082.s001.docx]

| **S1 Table. Spearman's correlation coefficients for the association between the levels of antimicrobial peptides in BAL and lung function. The association with smoking and inhaled steroid use (COPD only) was explored by Kruskal-Wallis tests.** | | | | | | | |
| --- | --- | --- | --- | --- | --- | --- | --- |
|  |  | SLPI | | hBD-1 | | hBD-2 | |
|  |  | *rho* | *p* | *rho* | *p* | *rho* | *p* |
|  | FVC % predicted | -0.7 | 0.02 | -0.2 | 0.6 | -0.4 | 0.2 |
|  | FEV_1_ % predicted | -0.6 | 0.047 | -0.3 | 0.4 | -0.2 | 0.6 |
| **IPF** | DLCO % predicted | 0.03 | 0.9 | 0.5 | 0.1 | -0.7 | 0.03 |
|  | Smoking |  | 0.4 |  | 0.3 |  | 0.9 |
|  | FVC % predicted | 0.2 | 0.6 | -0.1 | 0.7 | -0.2 | 0.5 |
|  | FEV_1_ % predicted | -0.5 | 0.09 | -0.4 | 0.2 | -0.3 | 0.4 |
| **COPD** | DLCO % predicted | -0.03 | 1.0 | 0.6 | 0.2 | 0.5 | 0.3 |
|  | Smoking |  | 0.8 |  | 0.1 |  | 0.5 |
|  | Inhaled steroid use |  | 0.06 |  | 0.6 |  | 0.3 |
|  | FVC % predicted | 0.08 | 0.8 | -0.05 | 0.9 | -0.4 | 0.2 |
|  | FEV_1_ % predicted | -0.07 | 0.8 | -0.4 | 0.2 | -0.1 | 0.7 |
| **Controls** | DLCO % predicted | 0.3 | 0.6 | -0.6 | 0.2 | -0.4 | 0.4 |
|  | Smoking |  | 0.2 |  | 0.8 |  | 0.8 |
|  | FVC % predicted | 0.09 | 0.6 | 0.2 | 0.3 | -0.5 | 0.001 |
|  | FEV1 % predicted | -0.2 | 0.3 | -0.3 | 0.04 | -0.3 | 0.1 |
| **Combined** | DLCO % predicted | 0.2 | 0.3 | 0.2 | 0.2 | 0.2 | 0.2 |
|  | Smoking |  | 0.7 |  | 0.02 |  | 0.9 |
|  | Inhaled steroid use |  | 0.04 |  | 0.07 |  | 0.3 |
