# Supplementary material for: The lower airways microbiome and antimicrobial peptides in idiopathic pulmonary fibrosis differ from chronic obstructive pulmonary disease
Source: PLoS One. 2022 Jan 6;17(1):e0262082. doi: 10.1371/journal.pone.0262082 (PMC8735599; doi:10.1371/journal.pone.0262082)
Supplement: S2 Table — For the categorical variables smoking (never, ex, current), and use of inhaled steroids (in COPD only), the correlation was assessed by Kruskal-Wallis tests, and p-values provided. (DOCX) [file pone.0262082.s002.docx]

| **S2 Table. Spearman's correlation coefficients and p-values between the lung function and the three AMPs stratified by study group, and the relative abundance (%) of the two most common phyla and three most common genera. For the categorical variables smoking (never, ex, current), and use of inhaled steroids (in COPD only), the correlation was assessed by Kruskal-Wallis tests, and p-values provided.** | | | | | | | | | | | | |
| --- | --- | --- | --- | --- | --- | --- | --- | --- | --- | --- | --- | --- |
| 2 |  | **Phylum** | | | | **Genus** | | | | | | |
|  |  | Firmicutes | | Bacteroidetes | | Streptococcus | | Prevotella | | Veillonella | | |
|  |  | *rho* | *p* | *rho* | *p* | *rho* | *p* | *rho* | *p* | *rho* | *p* |  |
|  | FVC % predicted | -0.04 | 0.9 | -0.02 | 0.9 | -0.3 | 0.3 | 0.3 | 0.4 | 0.7 | 0.02 |  |
|  | FEV1 % predicted | 0.2 | 0.5 | 0.3 | 0.4 | 0.1 | 0.6 | 0.6 | 0.04 | 0.6 | 0.04 |  |
|  | DLCO % predicted | -0.2 | 0.5 | -0.1 | 0.7 | -0.6 | 0.04 | -0.07 | 0.8 | 0.2 | 0.6 |  |
| **IPF** | SLPI | -0.4 | 0.3 | -0.1 | 0.7 | -0.09 | 0.8 | -0.4 | 0.2 | -0.9 | 0.0008 |  |
|  | hBD-1 | -0.6 | 0.03 | 0.3 | 0.4 | -0.8 | 0.007 | 0.1 | 0.8 | -0.2 | 0.6 |  |
|  | hBD-2 | 0.5 | 0.2 | -0.1 | 0.8 | 0.5 | 0.2 | 0.2 | 0.6 | -0.7 | 0.7 |  |
|  | Smoking |  | 0.2 |  | 0.3 |  | 0.3 |  | 0.2 |  | 0.3 |  |
|  | FVC % predicted | -0.4 | 0.2 | 0.4 | 0.2 | -0.4 | 0.2 | 0.3 | 0.3 | -0.2 | 0.6 |  |
|  | FEV1 % predicted | 0.3 | 0.3 | -0.2 | 0.5 | 0.2 | 0.5 | -0.1 | 0.7 | -0.5 | 0.1 |  |
|  | DLCO % predicted | -0.3 | 0.6 | 0.3 | 0.5 | -0.5 | 0.3 | 0.3 | 0.5 | 0.4 | 0.5 |  |
| **COPD** | SLPI | -0.3 | 0.3 | 0.5 | 0.1 | -0.5 | 0.1 | 0.3 | 0.4 | 0.4 | 0.2 |  |
|  | hBD-1 | 0.2 | 0.5 | 0.04 | 0.9 | -0.2 | 0.5 | -0.1 | 0.7 | 0.6 | 0.03 |  |
|  | hBD-2 | -0.09 | 0.8 | -0.2 | 0.5 | 0.4 | 0.2 | -0.4 | 0.2 | -0.1 | 0.8 |  |
|  | Smoking |  | 0.3 |  | 0.3 |  | 0.3 |  | 0.4 |  | 0.2 |  |
|  | Inhaled steroid use |  | 0.3 |  | 0.3 |  | 0.4 |  | 0.1 |  | 0.6 |  |
|  | FVC % predicted | 0.3 | 0.4 | -0.2 | 0.6 | 0.4 | 0.2 | -0.4 | 0.1 | -0.3 | 0.3 |  |
|  | FEV1 % predicted | 0.2 | 0.5 | -0.04 | 0.9 | 0.3 | 0.3 | -0.2 | 0.5 | 0.04 | 0.9 |  |
|  | DLCO % predicted | -0.2 | 0.7 | 0.4 | 0.4 | -0.1 | 0.8 | 0.009 | 1.0 | -0.2 | 0.6 |  |
| **Controls** | SLPI | -0.5 | 0.1 | 0.3 | 0.4 | -0.2 | 0.4 | 0.2 | 0.5 | -0.06 | 0.8 |  |
|  | hBD-1 | 0.05 | 0.9 | -0.07 | 0.8 | 0.1 | 0.7 | -0.2 | 0.6 | -0.2 | 0.4 |  |
|  | hBD-2 | -0.1 | 0.7 | 0.2 | 0.5 | -0.3 | 0.3 | 0.3 | 0.3 | 0.4 | 0.2 |  |
|  | smoking |  | 1.0 |  | 0.1 |  | 0.5 |  | 0.4 |  | 0.2 |  |
|  | FVC % predicted | 0.09 | 0.6 | 0.1 | 0.5 | 0.1 | 0.5 | -0.01 | 1.0 | -0.1 | 0.5 |  |
|  | FEV1 % predicted | 0.02 | 0.9 | 0.3 | 0.1 | -0.07 | 0.7 | 0.4 | 0.02 | 0.1 | 0.5 |  |
|  | DLCO % predicted | -0.009 | 1.0 | 0.2 | 0.3 | -0.2 | 0.3 | 0.2 | 0.3 | -0.01 | 1.0 |  |
| **All subjects** | SLPI | -0.2 | 0.2 | 0.1 | 0.4 | -0.2 | 0.4 | -0.1 | 0.4 | -0.1 | 0.4 |  |
|  | hBD-1 | 0.1 | 0.6 | -0.1 | 0.5 | 0.1 | 0.6 | -0.3 | 0.1 | -0.07 | 0.7 |  |
|  | hBD-2 | -0.1 | 0.4 | -0.08 | 0.6 | 0.006 | 1.0 | 0.03 | 0.9 | 0.04 | 0.8 |  |
|  | smoking |  | 0.5 |  | 0.03 |  | 0.3 |  | 0.03 |  | 0.4 |  |
