# Supplementary material for: The lower airways microbiome and antimicrobial peptides in idiopathic pulmonary fibrosis differ from chronic obstructive pulmonary disease
Source: PLoS One. 2022 Jan 6;17(1):e0262082. doi: 10.1371/journal.pone.0262082 (PMC8735599; doi:10.1371/journal.pone.0262082)
Supplement: S3 Table — (DOCX) [file pone.0262082.s003.docx]

| **S3 Table. Spearman's correlation coefficients for the association between alpha diversity assessed by Faith's phylogenetic and Shannon's non-phylogenetic to the antimicrobial peptides for the different study groups and all subjects** | | | | | | | | | | | | | | | | |
| --- | --- | --- | --- | --- | --- | --- | --- | --- | --- | --- | --- | --- | --- | --- | --- | --- |
|  | **IPF** | | | | **COPD** | | | | **Controls** | | | | **All subjects** | | | |
|  | Faith | | Shannon | | Faith | | Shannon | | Faith | | Shannon | | Faith | | Shannon | |
|  | *rho* | *p* | *rho* | *p* | *rho* | *p* | *rho* | *p* | *rho* | *p* | *rho* | *p* | *rho* | *p* | *rho* | *p* |
| hBD-1 | -0.3 | 0.1 | -0.1 | 0.5 | -0.3 | 0.1 | 0.06 | 0.8 | -0.01 | 0.9 | 0.2 | 0.3 | -0.03 | 0.8 | -0.08 | 0.4 |
| hBD-2 | 0.4 | 0.04 | 0.3 | 0.1 | 0.1 | 0.6 | -0.2 | 0.3 | -0.2 | 0.4 | 0.2 | 0.4 | 0.03 | 0.8 | 0.08 | 0.5 |
| SLPI | -02 | 0.3 | -0.2 | 0.2 | 0.03 | 0.9 | 0.3 | 0.2 | -0.06 | 0.7 | 0.09 | 0.6 | -0.01 | 0.9 | -0.06 | 0.6 |
