# Supplementary material for: The lower airways microbiome and antimicrobial peptides in idiopathic pulmonary fibrosis differ from chronic obstructive pulmonary disease
Source: PLoS One. 2022 Jan 6;17(1):e0262082. doi: 10.1371/journal.pone.0262082 (PMC8735599; doi:10.1371/journal.pone.0262082)
Supplement: S4 Table — (DOCX) [file pone.0262082.s004.docx]

| **S4 Table. Beta diversity p-values between study groups for different sampling methods (4a), and between different sampling methods between study groups (4b)** | | | | | | | | |
| --- | --- | --- | --- | --- | --- | --- | --- | --- |
|  |  |  |  |  |  |  |  |  |
| **4a** |  |  |  |  | **4b** |  |  |  |
|  | **PBAL** | **rPSB** | **OW** |  |  | **IPF** | **COPD** | **Ctrl** |
| *Weighted UniFrac* |  |  |  |  | *Weighted UniFrac* |  |  |  |
| IPF vs COPD | 0.002 | 0.012 | 0.225 |  | PBAL vs OW | 0.620 | 0.001 | 0.008 |
| IPF vs Controls | 0.009 | 0.017 | 0.163 |  | PBAL vs rPSB | 0.045 | 0.469 | 0.060 |
| COPD vs Controls | 0.556 | 0.621 | 0.079 |  | OW vs rPSB | 0.021 | 0.001 | 0.001 |
| *Unweighted UniFrac* |  |  |  |  | *Unweighted UniFrac* |  |  |  |
| IPF vs COPD | 0.014 | 0.016 | 0.699 |  | PBAL vs OW | 0.012 | 0.001 | 0.002 |
| IPF vs Controls | 0.019 | 0.030 | 0.661 |  | PBAL vs rPSB | 0.077 | 0.200 | 0.209 |
| COPD vs Controls | 0.556 | 0.433 | 0.265 |  | OW vs rPSB | 0.002 | 0.001 | 0.001 |
| *Bray-Curtis* |  |  |  |  | *Bray-Curtis* |  |  |  |
| IPF vs COPD | 0.009 | 0.016 | 0.150 |  | PBAL vs OW | 0.011 | 0.160 | 0.099 |
| IPF vs Controls | 0.670 | 0.050 | 0.740 |  | PBAL vs rPSB | 0.980 | 0.974 | 0.653 |
| COPD vs Controls | 0.320 | 0.407 | 0.220 |  | OW vs rPSB | 0.090 | 0.010 | 0.002 |
