# Supplementary material for: The lower airways microbiome and antimicrobial peptides in idiopathic pulmonary fibrosis differ from chronic obstructive pulmonary disease
Source: PLoS One. 2022 Jan 6;17(1):e0262082. doi: 10.1371/journal.pone.0262082 (PMC8735599; doi:10.1371/journal.pone.0262082)
Supplement: S1 Appendix — (PDF) [file pone.0262082.s005.pdf]

Supporting information for the article: "The lower airways microbiome and antimicrobial peptides in Idiopathic Pulmonary Fibrosis differ from Chronic Obstructive Pulmonary Diseases"

Evaluation of negative control samples

For each participant in this study, an oral wash (OW) sample was taken from the oral cavity, a protected bronchoalveolar lavage (PBAL) sample was taken from the middle lobe, an three protected sterile brushes were taken from the right lower lobe, all from the same location in the same subsegment (rPSB). Sterile PBS-buffered saline fluid was used for the OW gargling and instillation of the 100-150 mL of BAL fluid, and for dissolving in the same aliquot the three sterile tips of the brushes cut with sterile scissors.

For each participant, aliquots of the PBS fluid used for that particular participant was also stored, negative control samples taken directly from the sterile PBS fluid bottle, and never contaminated from either the participant of the bronchoscopy procedure.

For each participants, all samples were DNA extracted, PCR amplified and sequenced together in the same batches or sequencing RUNs, thus all four samples were subject to the same batches of the same laboratory reagents, and the same handling the same day. Thus, any sequences found in those negative controls were purely contamination from either handling during sampling directly from the sterile PBS saline bottle or during laboratory analyses. Previous published analyses by our group, have shown that most all contamination in our clinical samples come from the laboratory reagent kits, and most often the DNA extraction kits [1]. That same publication also demonstrated the usefulness of the Decontam algorithm in R [2] for bioinformatic clean-up.

A list of the relative abundance of the 20 most common taxa in those negative control samples, collapsed to the species level when possible, is provided below. Note that these 20 taxa comprise a little more than 90% of taxa found in the negative controls. Many taxa were not assigned at the genus or species level, and those only assigned as kingdom_Bacteria_;_ may also represent human DNA. In any event, the most commonly found taxa at the family level belonged to *Burkholderiaceae*, of which the most common genera was *Ralstonia*, a known common contaminant. Note also the very low frequency of *Streptococci* in the negative controls; which was by far the most common genera in all biological samples.

**Taxonomic assignment**	**Mean relative frequency**	**Cumulative frequency**
k__Bacteria;p__Proteobacteria;c__Betaproteobacteria;o__Burkholderiales;f__Burkholderiaceae; g__Ralstonia;s__pickettii	18.1 %	18.1 %
k__Bacteria;p__Proteobacteria;c__Gammaproteobacteria;o__Enterobacterales;f__Enterobacteriaceae;g__Klebsiella;s__pneumoniae	17.1 %	35.1 %
k__Bacteria;p__Firmicutes;c__Negativicutes;__;__;__;__	13.7 %	48.8 %
k__Bacteria;p__Proteobacteria;c__Betaproteobacteria;o__Burkholderiales;f__Burkholderiaceae;g__Ralstonia;s__sp._HMT_406	9.6 %	58.4 %
k__Bacteria;p__Proteobacteria;c__Betaproteobacteria;o__Burkholderiales;f__Comamonadaceae;g__Delftia;s__acidovorans	6.0 %	64.4 %
k__Bacteria;p__Bacteroidetes;c__Chitinophagia;o__Chitinophagales;f__Chitinophagaceae;g__Segetibacter;s__aerophilus	3.8 %	68.1 %
k__Bacteria;p__Proteobacteria;c__Gammaproteobacteria;o__Pseudomonadales;f__Pseudomonadaceae;g__Pseudomonas;s__fluorescens	3.7 %	71.8 %
k__Bacteria;p__Proteobacteria;c__Betaproteobacteria;o__Burkholderiales;f__Burkholderiaceae;g__Burkholderia;s__cepacia	3.5 %	75.3 %
k__Bacteria;p__Proteobacteria;c__Betaproteobacteria;o__Burkholderiales;f__Burkholderiaceae;__;__	3.3 %	78.6 %
k__Bacteria;p__Proteobacteria;c__Alphaproteobacteria;o__Rhizobiales;f__Rhizobiaceae;g__Agrobacterium;s__tumefaciens	2.6 %	81.1 %
k__Bacteria;p__Proteobacteria;c__Alphaproteobacteria;o__Caulobacterales;f__Caulobacteraceae;g__Brevundimonas;s__diminuta	1.9 %	83.1 %
k__Bacteria;__;__;__;__;__;__	1.4 %	84.5 %
k__Bacteria;p__Proteobacteria;c__Gammaproteobacteria;__;__;__;__	1.1 %	85.6 %
k__Bacteria;p__Proteobacteria;c__Alphaproteobacteria;o__Rhizobiales;__;__;__	0.9 %	86.5 %
k__Bacteria;p__Proteobacteria;c__Gammaproteobacteria;o__Pseudomonadales;f__Pseudomonadaceae;g__Pseudomonas;__	0.8 %	87.4 %
k__Bacteria;p__Proteobacteria;c__Alphaproteobacteria;o__Rhizobiales;f__Bradyrhizobiaceae;__;__	0.8 %	88.2 %
k__Bacteria;p__Proteobacteria;c__Alphaproteobacteria;o__Rhizobiales;f__Brucellaceae;g__Ochrobactrum;s__anthropi	0.8 %	89.0 %
k__Bacteria;p__Firmicutes;c__Bacilli;o__Lactobacillales;f__Streptococcaceae;g__Streptococcus;__	0.7 %	89.7 %
k__Bacteria;p__Actinobacteria;c__Actinobacteria;o__Actinomycetales;f__Micrococcaceae;g__Micrococcus;s__luteus	0.6 %	90.3 %
k__Bacteria;p__Firmicutes;c__Negativicutes;o__Veillonellales;f__Veillonellaceae;g__Veillonella;s__parvula	0.6 %	90.9 %

One may ask what impact the Decontam removal of contaminants have had on the taxonomic assignment in the current study.

A list of the 25 most common genera found in BAL fluid for our 36 study participants before and after Decontam answers this question:

First before:

Genera	Mean relative frequency
Streptococcus	24.2 %
Veillonella	8.9 %
Prevotella	8.3 %
Klebsiella	6.7 %
Ralstonia	6.6 %
Unassigned	5.6 %
Rothia	5.1 %
Unassigned Negativicutes	3.1 %
Gemella	2.5 %
Haemophilus	2.5 %
Neisseria	2.1 %
Porphyromonas	1.8 %
Granulicatella	1.8 %
Leptotrichia	1.8 %
Delftia	1.5 %
Actinomyces	1.3 %
Megasphera	1.2 %
Fusobacterium	1.2 %
Saccharibacteria_(TM7)_[G-1]	1.1 %
Selenomonas	1.1 %
Moraxella	1.0 %
Pseudomonas	0.9 %
Capnocytophaga	0.8 %
Campylobacter	0.8 %
Segetibacter	0.7 %

And then mean relative frequency in BAL for all 36 participants after Decontam removal of contaminants:

Genera	Mean relative frequency
Streptococcus	34.1 %
Veillonella	11.3 %

Unassigned	10.9 %
Prevotella	9.2 %
Rothia	3.8 %
Gemella	3.7 %
Granulicatella	3.2 %
Leptotrichia	2.4 %
Porphyromonas	2.0 %
Saccharibacteria_(TM7)_[G-1]	1.7 %
Haemophilus	1.6 %
Megasphaera	1.6 %
Moraxella	1.5 %
Actinomyces	1.4 %
Campylobacter	1.3 %
Capnocytophaga	1.1 %
Selenomonas	1.1 %
Neisseria	1.0 %
Fusobacterium	1.0 %
Atopobium	0.8 %
Oribacterium	0.7 %
Alloprevotella	0.5 %
Lachnoanaerobaculum	0.4 %
Solobacterium	0.4 %
Klebsiella	0.4 %

Notably *Ralstonia* is removed, and *Klebsiella* massively reduced, whereas the relative frequency - and dominance - of *Streptococcus* becomes more apparent.

Evaluation of features influencing the vectors of the beta diversity PCoA plots

Loading plots were made in QIIME 2 with the biplot plugin of the emperor plot command, and are shown for IPF, COPD, and control subjects respectively:

Beta diversity in 12 IPF patients, by three different sample types; red dots = OW; blue dots PBAL; and orange dots rPSB.

The five most common ASVs (loadings) are shown by vectors; annotated by species level if possible, or else genus level. Two ASVs had the same taxonomic annotation at the species level (both R*othia aeria*).

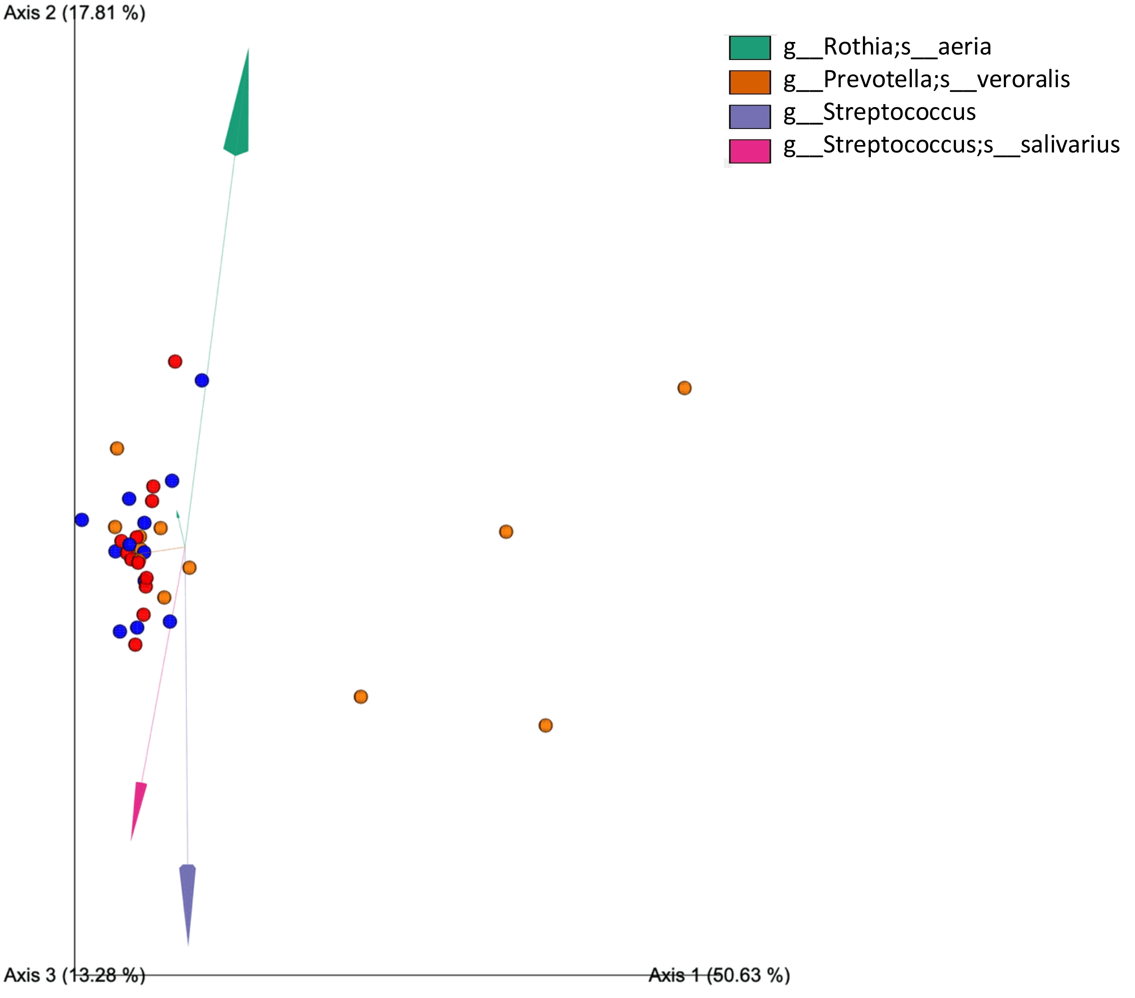

A similar plot shows the loadings for the 12 COPD patiens, with the same coloring of the sample types:

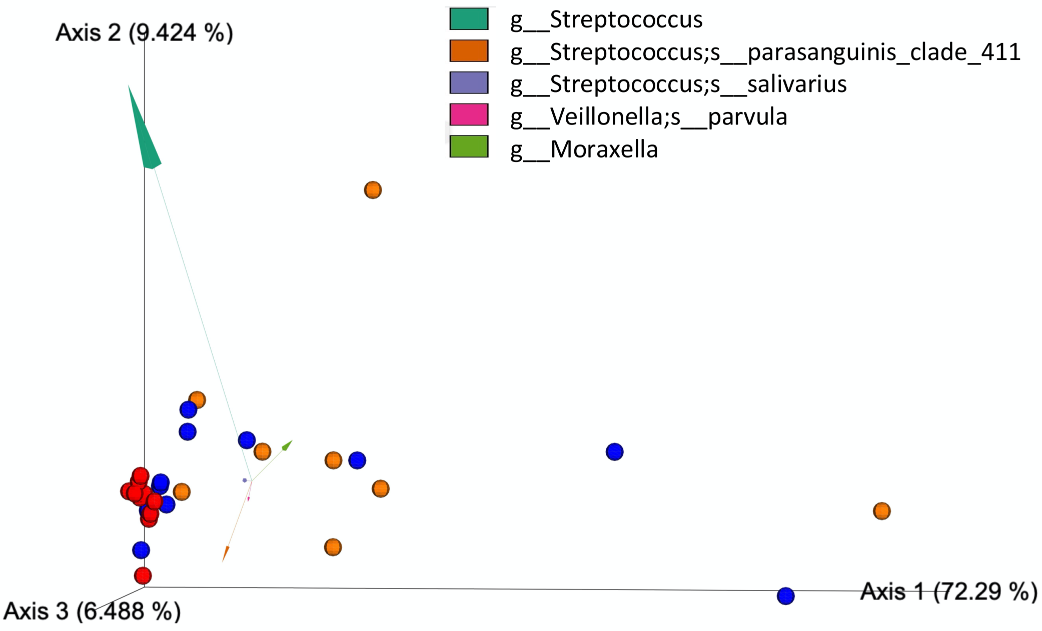

And finally, the same type of plot for the 12 healthy control subjects, again with the same coloring of the three sample types:

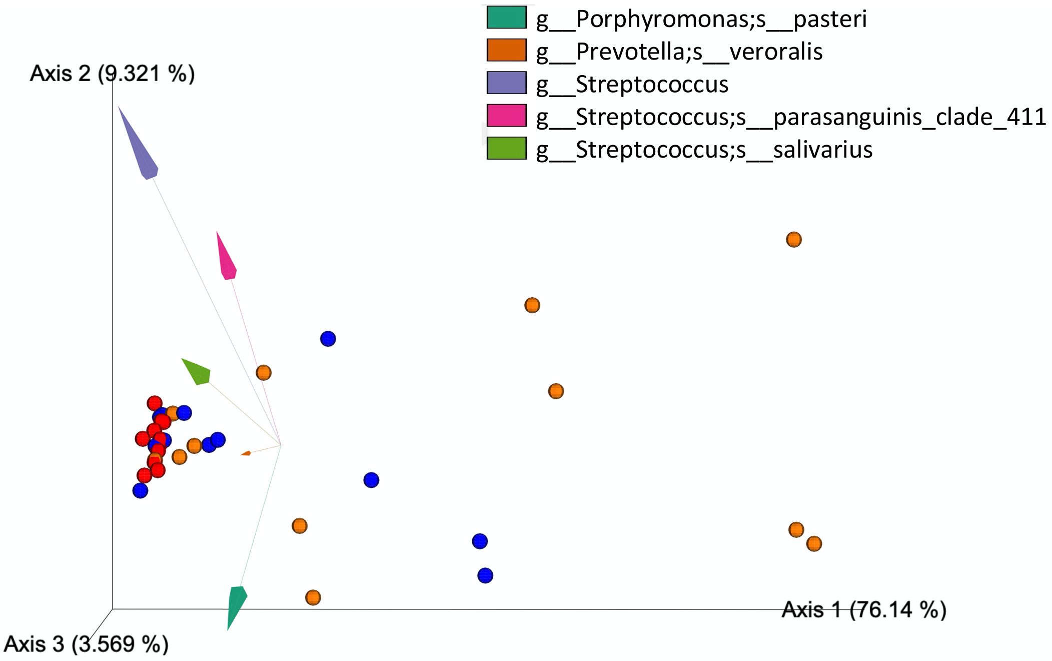

For all study groups, *streptococci* had a large influence on the beta diversity. The influence appeared more even among taxa for the control subjects than for especially the COPD patients, where one streptococcus genus (unclassified at the species level) appeared to have a large influence.

References

1. Drengenes C, Wiker HG, Kalananthan T, Nordeide E, Eagan TML, Nielsen R (2019) Laboratory contamination in airway microbiome studies. BMC Microbiol 19: 187.
2. Davis NM, Proctor DM, Holmes SP, Relman DA, Callahan BJ (2018) Simple statistical identification and removal of contaminant sequences in marker-gene and metagenomics data. Microbiome 6: 226.
